# Lowered Delta Activity in Post-COVID-19 Patients with Fatigue and Cognitive Impairment

**DOI:** 10.3390/biomedicines11082228

**Published:** 2023-08-08

**Authors:** Paola Ortelli, Angelica Quercia, Antonio Cerasa, Sabrina Dezi, Davide Ferrazzoli, Luca Sebastianelli, Leopold Saltuari, Viviana Versace, Angelo Quartarone

**Affiliations:** 1 Department of Neurorehabilitation, Hospital of Vipiteno (SABES-ASDAA), Teaching Hospital of the Paracelsus Medical Private University (PMU), 39049 Vipiteno-Sterzing, Italy; 2 Department of Clinical Psychology, Hospital of Bressanone (SABES-ASDAA), Teaching Hospital of the Paracelsus Medical Private University (PMU), 39049 Vipiteno-Sterzing, Italy; 3 Department of Biomedical, Dental, Morphological and Functional Imaging Sciences, University of Messina, 98122 Messina, Italy; 4 Institute for Biomedical Research and Innovation (IRIB), National Research Council of Italy (CNR), 98164 Messina, Italy; 5 Severe Acquired Brain Injury Unit, S’Anna Institute, 88900 Crotone, Italy; 6 Pharmacotechnology Documentation and Transfer Unit, Preclinical and Translational Pharmacology, Department of Pharmacy, Health and Nutritional Sciences, University of Calabria, 87036 Rende, Italy; 7 IRCCS Centro Neurolesi “Bonino Pulejo”, 98123 Messina, Italy

**Keywords:** post-COVID syndrome, fatigue, cognitive impairment, Hd-EEG, eLORETA

## Abstract

In post-COVID-19 syndrome (PCS), neurocognitive symptoms and fatigue are often associated with alterations in electroencephalographic (EEG) activity. The present study investigates the brain source activity at rest in PCS patients (PCS-pts) perceiving cognitive deficits and fatigue. A total of 18 PCS-pts and 18 healthy controls (HCs) were enrolled. A Montreal Cognitive Assessment (MoCA), Perceived Cognitive Difficulties Scale (PDCS) and Fatigue Severity Scale (FSS) were administered for assessing the symptoms’ severity. Brain activity at rest, both with open (OE) and closed eyes (CE), was recorded by high-density EEG (Hd-EEG) and localized by source estimation. Compared to HCs, PCS-pts exhibited worse performance in executive functions, language and memory, and reported higher levels of fatigue. At resting OE state, PCS-pts showed lower delta source activity over brain regions known to be associated with executive processes, and these changes were negatively associated with PDCS scores. Consistent with recent literature data, our findings could indicate a dysfunction in the neuronal networks involved in executive functions in PCS-pts complaining of fatigue and cognitive impairment.

## 1. Introduction

The World Health Organization (WHO) (https://www.nice.org.uk/guidance/ng188, accessed on 30 August 2022) has recently updated the informal term for a combination of symptoms that appear or last longer than 4 weeks after the first COVID-19 infection and cannot be adequately explained by another diagnosis as post-COVID-19 syndrome (PCS) [1]. The involvement of the nervous system represents an important component of post-COVID-19 syndrome, better known as neuro-long COVID [2].

Cognitive impairment and fatigue are the most frequent and debilitating long-lasting neurological symptoms complained of with PCS, even after mild or moderate infection [3]. Cognitive impairment is very often associated with fatigue, associated with a plethora of symptoms that could be interpreted as “brain fog” [4]. This last label has been coined outside the scientific context by patients that first developed PCS-related neurocognitive symptoms. In fact, during the first part of the pandemic, researchers focused only on severe COVID-19 conditions and their sequelae. Nowadays, PCS-related neurocognitive symptoms represent one of the most frequent and alarming sequelae of this disease because of their dramatic incidence in the general population. No “universally accepted” definition of “brain fog” has yet been established [4]; additionally, no specific neuropsychological assessment has been defined [3].

In PCS patients (PCS-pts), previous studies reported specific deficits in sustained and selective attention, abstraction, inhibition, set shifting, learning and long-term memory [3,5,6]. Part of these deficits may be related to intrinsic aspects of fatigue; in fact, lack of energy, muscle weakness, as well as global slowing in reaction times, could ultimately impact executive functions [3,7,8,9].

Currently, the neural basis underlying cognitive deficits and fatigue in PCS is not yet fully understood. Previous studies have indicated that the neural basis of cognitive impairment and fatigue in PCS may involve a combination of factors, including neuroinflammation, immune system dysfunctions and dysregulation of the autonomic nervous system [10,11,12]. Neuroinflammation, triggered by the binding of the SARS-CoV-2 virus to angiotensin-converting enzyme 2 (ACE2) receptors in the brain, leads to a cytokine storm and activation of microglia, astrocytes and oligodendrocytes. This inflammatory response can result in a reduction in myelinated axons, impairment of hippocampal neurogenesis and overall disruption of neural circuit functions, thereby contributing to cognitive impairment [10,12,13,14,15,16,17,18,19]. It is known that an imbalance between pro-inflammatory and anti-inflammatory factors could represent a possible basis for understanding the pathophysiology of cognitive impairment, together with alterations in brain activity, in several chronic diseases (as has been demonstrated, for example, in migraine and neuropathic pain). Therefore, it is conceivable that chronic inflammation in PCS-pts “stresses” the neuronal activities, leading in turn to brain network dysfunctions and cognitive impairment [19]. Additionally, functional abnormalities have been observed in various brain regions, including the frontal lobe structures, olfactory cortices, limbic system and prefrontal cortex [13,14,15,16,17,18]. These regions are known to be involved in attention, memory, executive functions and emotional processing.

Electroencephalography (EEG) studies have also provided insights into the neurophysiological impact of SARS-CoV-2 infection, revealing changes in brain electrical activity that correlate with cognitive deficits in PCS patients [17,20,21,22,23,24,25]. Investigating the neural substrates of cognitive deficits and fatigue in PCS patients can provide valuable insights into the pathophysiology of this condition and open the way to the development of targeted interventions for affected individuals. The EEG patterns of PCS-pts were evaluated with different types of analyses, from common power spectrum and event-related potentials (ERPs) [17,20,22] to more complex brain dynamic analyses, such as intrinsic mode functions (IMFs) and avalanche analysis [21,23]. The excellent temporal resolution of EEGs is suitable for brain dynamic analysis. However, the aforementioned analyses are performed at scalp level, and therefore, they do not permit for estimation of which brain areas generated the electric signals, a fundamental aspect to understanding pathophysiological mechanisms in the brain. In PCS patients, Cecchetti and colleagues [17] performed, instead, a source estimation analysis by using a low-density EEG. This technique is more accessible in clinical settings than high-density EEG (Hd-EEG), but it implies a low spatial resolution that could potentially flaw the source localization analysis as compared to high-density source estimation [26].

In this study, we sought to investigate the sources of brain alterations in patients suffering from PCS complaining of “brain fog” (cognitive impairment and fatigue) by using high-density EEG at rest.

## 2. Materials and Methods

### 2.1. Participants

The study was conducted at the “Post COVID” outpatient clinic of the Department of Neurorehabilitation (Hospital of Vipiteno, SABES-ASDAA, Vipiteno-Sterzing, Italy) between March and October 2021. We enrolled patients that received a diagnosis of PCS, in accordance with WHO criteria, and complained of cognitive difficulties and/or fatigue. The following inclusion criteria had to be fulfilled to avoid situations in which cognitive disturbances and fatigue could be related to factors other than SARS-CoV-2 infection: (a) absence of neurological disorders prior to COVID-19; (b) absence of prior or current diagnosis of psychiatric, endocrine, metabolic or cardiopulmonary conditions related to fatigue; (c) absence of dyspnea or other long-lasting sequelae of interstitial COVID-19 pneumonia; (d) absence of anemia; (e) no treatment with corticosteroids or antihistaminic, antihypertensive, diuretic or hypnotic drugs at the time of the study; and (f) absence of hospitalization and/or ventilation in the acute phase. Eighteen patients fulfilled these criteria and were enrolled in the study (10 females; age range = 27.60–57.20 years, mean ± SD: 41.11 ± 9.07 years; education range = 7.0–17.0 years, mean ± SD: 14.5 ± 2.7 years; 17 right-handers out of 18). Eighteen healthy subjects were also recruited as controls (HCs), matched for age and gender (8 females, *p*-value = 0.06; age range = 25.39–87.81 years, mean ± SD: 48.28 ± 18.08 years, *p*-corrected = 0.53 vs. patients; education range = 8.0–17.0 years, mean ± SD: 14.11 ± 3.14 years, *p*-corrected = 0.83 vs. patients; all right-handers). HCs were enrolled among hospital personnel who underwent weekly SARS-CoV-2 screening tests by nasopharyngeal swabs.

All participants signed an informed written consent form for the use of their clinical data for scientific purposes. Demographic data, medical history and previous PCR test results were collected. All patients and HCs underwent an extensive neuropsychological and neurophysiological evaluation. The study was approved by the local Ethics Committee (“Comitato Etico del Comprensorio Sanitario di Bolzano”, 65–2020) in accordance with the Code of Ethics of the World Medical Association (Declaration of Helsinki, 1967).

### 2.2. Clinical Assessment

Demographic data (sex, age, years of education and handedness), medical history and previous PCR test results were collected.

Global cognition and executive functions were evaluated with the Montreal Cognitive Assessment (MoCA), a rapid screening test designed to investigate cognitive profiles. The assessment include attention, concentration, executive function, memory, language, visuospatial and orientation tasks [27]. Moreover, to evaluate the severity of cognitive difficulties in daily living, patients were asked to rate their perceived cognitive difficulties over the week preceding the evaluation with the Perceived Cognitive Difficulties Scale (PCDS) by referring to one or more of the following symptoms: forgetfulness, cloudiness, difficulty focusing, thinking and communicating. The PCDS score is based on a 4-point Likert scale: 0 = “I have no cognitive difficulties”; 1 = “I have slightly more cognitive difficulties than before COVID”; 2 = “I have moderate cognitive difficulties most of the time”; 3 = “I have persistent cognitive difficulties” [7,8].

Fatigue was assessed in both PCS-pts and healthy controls (HC) with the Fatigue Severity Scale (FSS), one of the most frequently used questionnaires to measure fatigue in people with different chronic conditions and disorders. The scale consists of 9 sentences related to the interference of fatigue with daily functioning in different contexts, from family and work to social life and physical activity, rated on the basis of the degree of perceived severity on a 7-point scale (1 = “strongly disagree”; 7 = “strongly agree”). A total score of 36 or more suggests a high level of perceived fatigue that should be evaluated by a physician [28].

### 2.3. EEG Recording and Analysis

For each group (PCS-pt and HC), 3 min of resting state with eyes open (EO_RS) and eyes closed (EC_RS) were recorded using high-density EEG (Hd-EEG). Hd-EEG signals were collected using the 64-channel actiCap System (Brain Products GmbH, Munich, Germany) with electrodes positioned at the sites of the 10-10 EEG International System, with a sampling frequency of 5 kHz and referenced to the FCz electrode. Electrolyte gel was used to attain conductivity between the skin and the electrodes, keeping all impedances below 5 kΩ. Off-line signal processing was performed using the EEGlab toolbox (v.2021.0) [29] for MATLAB (Mathworks Inc., Natick, MA, USA, v. 2019a). Data were band-pass filtered (Finite Impulse Response Filter; 0.5–45 Hz; notch at 50 Hz) and down-sampled at 1000 Hz. Each recording was visually inspected to identify and remove channels and epochs containing sporadic artifacts. After the rejection of bad channels and epochs, we performed an independent component analysis (ICA) using FastICA, a toolbox for EEGlab, to identify stereotypical artifacts (i.e., ocular, musclar and electrocardiographic artifacts). Only ICA components with specific activity patterns and component maps characteristic of artefactual activity, as flagged by the ICLabel plugin [30], were removed after a visual classification. At the end of preprocessing, in order to perform the source estimation for each subject, bad channels were excluded. Therefore, for each subject, good channels were averaged and referenced, and artifact-free data were fragmented into epochs of 4 s to proceed to source localization analysis.

### 2.4. Source Localization

EEG signals at scalp level cannot be associated directly with the brain areas from which the signals originate, because each sensor measures signal contributions from different sources due to volume spread, which leads to source mixing. Therefore, to localize the sources of brain activity, a current density analysis was performed in three-dimensional (3D) MNI space using the exact low-resolution brain electromagnetic tomography, eLORETA software (v20200709; LORETA, Werribee, Australia), in the frequency domain [31]. To estimate current source density (CSD, μA/mm^2^) values at any cortical voxel, the eLORETA algorithm solves the EEG inverse problem with a zero-localization error solution, despite structured noise. For the inverse solution, eLORETA constructs a spatial transformation matrix, co-registering EEG electrodes to a 3-shell spherical head model, including the brain, the skull and scalp compartments, based on the digitized MRI from the Brain Imaging Centre at the Montreal Neurological Institute (MNI). The solution space was restricted to the cortical gray matter and the CSD is estimated on a grid of 6239 voxels, with a spatial resolution of 5 mm. In the present study, the exact 3D position of each electrode was not digitalized; therefore, the standard 10-10 EEG International System of electrode positions was used. After the computation of the transformation matrix for each subject, EEG cross-spectra were calculated using the Fast Fourier Transform algorithm (4 s of interval) for the subsequent frequency bands: delta (0.5–3.5 Hz), theta (4–7.5 Hz), alpha (8–12 Hz), beta 1 (13–18 Hz), beta 2 (18.5–21 Hz), beta 3 (21.5–30 Hz) and gamma (30.5–45 Hz). For each frequency band, then, the 3D cortical distribution of brain sources was computed.

Within each frequency band, comparisons of the current density distribution between PCS-pts and HCs, for both conditions (EO_RS and EC_RS), were evaluated using the statistical nonparametric mapping (SnPM) methodology with correction for multiple comparisons, available in the eLORETA software. Specifically, the difference in current density distribution between PCS-pts and HCs, for each condition and frequency band, was assessed by voxel-by-voxel independent sample F-ratio tests, based on eLORETA log-transformed current density power. Significant differences in source voxels were identified by non-parametric voxel-wise randomization tests (5000 permutations, Fisher’s permutation method; threshold at the 5% probability level). The mean source power of each voxel and the distribution of the permuted values were compared and corrected for multiple comparisons across all voxels and all frequencies. Cortical maps of the differences between PCS-pts and HCs for each condition and frequency band were obtained. For each significant voxel, the eLORETA software provided the MNI coordinates, the lobe and the Brodmann area (BA). For each region of interest, the coordinates of the voxel corresponding to the maximum value and associated source power were found with in-house MATLAB scripts.

### 2.5. Statistical Analyses

Data analysis was performed via R-coded scripts (R version 4.0.5), using R packages (freeware and packages available for free on https://cran.r-project.org, accessed on 31 March 2021). Outliers were defined as data points below Q1 − 1.5 ∗ IQR (interquartile range) or above Q3 + 1.5 ∗ IQR. No subjects were excluded from all statistical analyses. Outlier influence diagnostics were performed before data analysis. Outliers were replaced with missing values (NA) and then predicted by imputation treatment implemented in the Mice R-Package [32]. No subjects were excluded from the statistical analysis. Due to the skewness of the distributions of the data, nonparametric tests were performed. A Mann–Whitney U-test was performed to compare neuropsychological data between PCS-pts and HCs, except for gender data, which were compared with the Chi-square test (ꭕ^2^). Moreover, nonparametric Spearman’s rho correlations were used to correlate significant changes in source estimation power (maximum value at voxel level) with the neuropsychological data of PCS-pts. Both *t*-tests and correlations were corrected for multiple comparisons (Benjamini–Hochberg correction, BH).

## 3. Results

### 3.1. Clinical Assessment

Demographic and clinical data of PCS-pts and HCs are displayed in Table 1. Females and males were equally represented in our sample. Eighty-nine percent of patients were less than 65 years old, and 99.0% were right-handed. PCS-pts and HCs did not differ significantly in age, education and gender (Table 1). The average time from onset (the elapsed interval between COVID-19 infection and study enrollment) was 189.5 (115.5) days, with 22.22% of cases exceeding six months since the infection.

Neurophysiological results are reported in Table 1. In regard to global cognition, MoCA tests revealed significantly lower performance in PCS-pts compared to HCs (W = 264, p_BH_-corrected < 0.01, r (rank biserial) = 0.63, 95% CI [0.34, 0.81], n_obs_ = 36). Analyzing the MoCA subscores in each specific cognitive domain, significant differences between PCS-pts and HCs emerged for executive functions, language and memory (W = 266, p_BH_-corrected < 0.001, r (rank biserial) = 0.64, 95% CI [0.36, 0.82], n_obs_ = 36; W = 278, *p*-adjusted < 0.001; W = 278, p_BH_-corrected = 0.048, r (rank biserial) = 0.72, 95% CI [0.48, 0.86], n_obs_ = 36; respectively). Visuospatial functions, orientation and attention did not differ significantly. The self-evaluation scale measuring perceived fatigue (FSS) revealed significantly higher scores in PCS-pts than in HCs (W = 0, p_BH_-corrected < 0.001, r (rank biserial) = −1.00, 95% CI [−1.00, −1.00], n_obs_ = 36).

No association emerged among FSS, PCDS and MoCA scores. No associations emerged between the time elapsed from disease onset and the neuropsychological and/or neurophysiological variables.

### 3.2. Source Analysis

The statistical analysis of current source density revealed significant differences between PCS-pts and HCs in the delta frequency band (mean CSDs: PCS-pts: 1.18, HCs: 5.86; 142 voxels), with PCS-pts exhibiting reduced activity compared to HCs (log-F-ratio threshold = −2.156, *p*-corrected = 0.0006, one-tailed). For the sake of clarity, there was also a tiny reduction in theta activity in PCS-pts compared to HCs (mean CSD: PCS-pts: 0.47, HCs: 1.57; 3 voxels). No other significant differences in source localization were found between PCS-pts and HCs with respect to the alpha, beta1, beta2, beta3 and gamma frequency bands. Only the voxels whose values exceeded the 97% percentile of the value distribution were considered to individuate cortical regions of significant activity.

**Figure 1:**
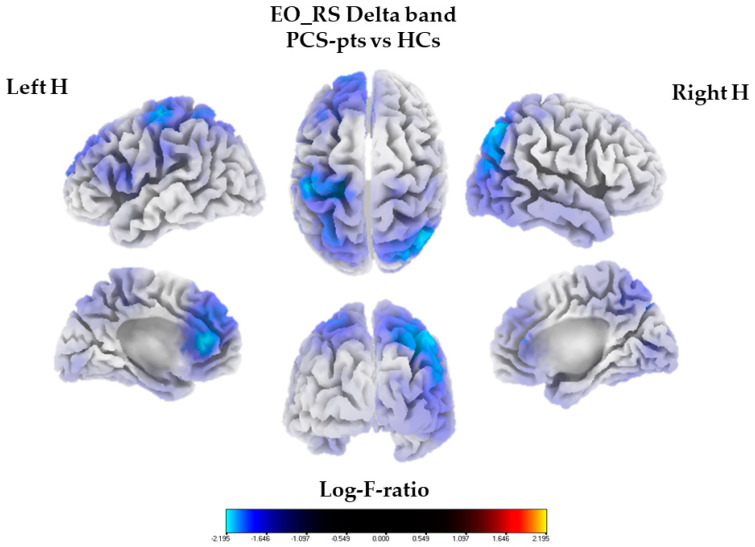
Three-dimensional eLORETA statistical maps of the delta frequency band (0.5–3.5 Hz). Colored areas represent the spatial extent of voxels with a significant difference between post-COVID-19 patients and healthy controls in source current density (*p* < 0.01, corrected). Significant results are projected onto a fiducial cortical surface. The color scale represents log-F-ratio values (threshold: log-F = −2.156, *p*-corrected < 0.001).

As shown in Figure 1, in PCS-pts, reduced delta activity was distributed bilaterally over the frontal–parietal lobe and in the left temporal lobe, with the postcentral gyrus showing the highest current density difference (x = −35, y = −25, z = 50; log-F-ratio = −2.414, *p*-corrected = 0.0006, one-tailed, Table 2). For each cortical region of significant activity, the coordinates of the voxel corresponding to the maximum value were obtained, as shown in Table 2. As for delta activity, differences between PCS-pts and HCs were found in the subsequent regions of interest: the left anterior cingulate, the left precentral gyrus, the left medial frontal gyrus, the right inferior parietal lobule, the right superior parietal lobule, the right precuneus, the middle temporal gyrus and the right angular gyrus (log-F-ratio threshold = −2.173, *p*-corrected = 0.0004, one-tailed). Furthermore, in PCS-pts, the maximum values of delta power at the voxel level of the left precentral gyrus, the left postcentral gyrus and the right superior parietal lobule were negatively related with PDCS scores (ρ = −0.52, 95% CI [−0.80, −0.06], S = 1477.17, p_BH_-corrected = 0.036; ρ = −0.50, 95% CI [−0.79, −0.03], S = 1455.47, p_BH_-corrected = 0.045; ρ = −0.56, 95% CI [−0.82, −0.03], S = 1511.65, p_BH_-corrected = 0.023; respectively).

## 4. Discussion

In the present study, we demonstrated changes in brain source activity at rest in PCS-pts complaining of cognitive deficits and fatigue. In line with previous studies, we found that executive functions, memory and language were defective in PCS-pts as compared to HCs [3,5,6,7,9]. Interestingly, the global cognitive score was normal [27], thus implying that, overall, PCS-pts did not have clinical, significant cognitive impairment. Lower performance in cognitive tasks, especially in those evaluating executive function, was associated with brain activity changes in PCS patients, both in classical EEG analyses (power spectra and ERPs) and brain dynamic analyses (intrinsic mode functions and avalanches) [21,23]. Wojcik and colleagues [21] found significant changes in brain dynamics in PCS patients during a task-switching experiment, as well as differences in ERP analyses between the “brain fog” group and the control group (post-COVID patients without “brain fog” and healthy subjects). Moreover, Appelt and colleagues [23] found an abnormal modulation of EEG activity in PCS-pts during the Trail Making Tests (TMT), associated with an increase in the time of execution. In particular, they found EEG changes over frontal regions, which are involved in executive function. However, EEG complexity also decreased in PCS-pts in a resting state [23].

Although there are substantial differences in the EEG methodology employed, our results are in line with these previous findings [20,22]. Indeed, we found a reduction in brain source activity at rest in the frontal, parietal and temporal brain regions when PCS-pts were compared to HCs. Of note, the main finding of our study is the reduction in brain source activity for the delta band (0.5–3.5), distributed bilaterally in the frontal–parietal lobe, and in the left temporal lobe, with the postcentral gyrus having the highest current density difference. Similarly, Kopanska and colleagues [20] found a decrease in delta activity in the left hemisphere of PCS-pts compared to pre-COVID EEG activity in the same subjects. On the other hand, Cecchetti and colleagues [17] found lowered delta activity at baseline (2 months after acute infection) in PCS-pts, related to a lower performance in executive function tasks (i.e., TMT and FAB), predicting worse cognitive performances at follow-up (10 months). Coherently, among all cognitive processes related to delta oscillations (e.g., spatial navigation), it has been suggested that delta activity in the anterior regions of the brain, especially in the frontal cortex, may be related to the inhibition of interferences during attentional shifting tasks. Thus, fine tuning of delta activity in the frontoparietal network is essential to reach better performance in attentional cognitive tasks [33]. Higher GABA concentration in frontal cortex was associated with more efficient suppression of distractors [34]. In line with these findings, in the elderly, higher GABA concentration in the frontal regions has been associated with better cognitive performance on the MoCA [35]. We do not have direct evidence of a link between reduced delta activity and GABAergic dysfunction. Nevertheless, recently, a transcranial magnetic stimulation (TMS) study in PCS-pts complaining of fatigue found a link between impairment in executive function and GABAergic inhibition [36]. Specifically, PSC patients showed an alteration of short- and long-interval intracortical inhibition (SICI and LICI), which may reflect a reduction in intracortical GABAergic activity in the primary motor cortex (M1). These neurophysiological findings highlight the presence of central motor and cognitive fatigue associated with GABAergic circuit dysfunction in PSC-pts [7,8,9,36]. GABAergic neurons are known to have a high expression of ACE2 receptors [37,38], known to be SARS-CoV-2 targets [12,39]. Therefore, in PCS-pts, the neuroinflammation may induce central GABAergic impairment, representing a common denominator for motor and cognitive fatigue and executive deficits.

Taken together, these results support the hypothesis that a dysfunction in the GABAergic system is involved in the pathophysiology of “brain fog” in PCS-pts [7,8,9,36]. In this study, “brain fog” was investigated by both FSS and PDCS. Our PCS-pts still perceived significant fatigue in daily life for a long time after the acute illness, as indicated by significantly higher FSS scores compared to HCs. Moreover, PCDS scores indicated that PCS-pts complain of high levels of perceived cognitive difficulties, in line with neuropsychological data. To our knowledge, this is the first study that investigated the relationship between EEG features and an objective measure of perceived cognitive difficulties. Intriguingly, PDCS scores were negatively correlated with delta source power. As previously explained, a reduction in delta activity has been associated with executive attentional deficits [34]. Recently, motor and somatosensory brain network changes were found in stroke patients in association with fatigue symptoms in EEG functional connectivity studies at rest [40,41]. In particular, Wu and colleagues [41] found a decrease in delta band activity in the left motor network. Similarly, in our PCS-pts, the abovementioned negative correlations between delta activity and the PDCS were found on the left precentral and postcentral gyrus, and on the right superior parietal lobule.

## 5. Conclusions and Future Directions

In conclusion, mild PCS-pts could manifest specific neuropsychological and neurophysiological features. They show objective, although subclinical, reductions in cognitive performance and higher fatigability as compared to HCs. These patients, at the same time, showed a reduction in delta source power. This finding is very intriguing. We hypothesize that PCS-pts, complaining of cognitive deficits, do not have a cognitive dysfunction so defective as to lead to clinical impairment. Nevertheless, these alterations affect their cognition. In line with these observations, the reduction in delta source activity at rest in PCS patients was found bilaterally in the frontal–parietal lobe network, and in the left temporal lobe; these are brain regions involved in the central executive network (CEN), default mode network (DMN), salience network (SN) and sensorimotor network (SMN). In the upcoming future, it would be interesting to examine in-depth changes of functional connectivity in PCS-pts in these networks.

## 6. Limitations

Our study has some limitations. First, we had neither neuropsychological nor neurophysiological data collected previous to the SARS-CoV-2 infection. Second, we did not perform a deep neuropsychological assessment; in our sample, that assessment was limited to MoCA. Further studies will broaden these results, taking into account these limitations. Finally, at the level of EEG analysis, we could not digitalize the electrodes’ positions and we did not have structural brain imaging of both HCs and PCS-pts; this additional information would have improved the source estimation analysis.

## Figures and Tables

**Table 1:** Comparison of demographic and clinical data between post-COVID-19 patients and healthy controls.

	PCS-pt	HC	*p*-Corrected
**Demographic data**			
Age (years)	41.11 ± 9.07	48.28 ± 18.08	0.53
Gender (female, *n*)	10 (55.56%)	8 (44.44%)	0.06
Education (years)	14.5 ± 2.7	14.11 ± 3.14	0.83
**Clinical assessment**			
Time from onset (days)	189.5 ± 115.5	-----	-----
**Fatigue evaluation**			
Perceived Cognitive Difficulties (PCDS) Range 0–3	1.66 ± 0.59	-----	-----
Fatigue Severity Scale (FSS) Range 0–63 (cut-off: 36)	44.22 ± 9.80	10.00 ± 1.90	**<0.001**
**Cognitive Assessment**			
Montreal Cognitive Assessment (MoCA) Range 0–30, cut-off 15.5	25.56 ± 2.25	28.25 ± 2.02	**0.005**
- Executive functions (sub-score)	2.94 ± 0.87	3.88 ± 0.32	**<0.001**
- Language (sub-score)	3.94 ± 1.05	5.38 ± 0.69	**<0.001**
- Memory (sub-score)	3.38 ± 1.33	4.33 ± 0.84	**0.048**
- Visuospatial (sub-score)	3.72 ± 0.75	3.77 ± 0.54	0.1
- Orientation (sub-score)	5.94 ± 0.23	6.00 ± 0.00	0.517
- Attention (sub-score)	5.67 ± 0.97	5.77 ± 0.54	1

Significant differences are reported in bold.

**Table 2:** Cortical regions of significant activity.

Eyes-Open Resting State (PCS-pts vs. HCs)					
		MNI Coordinates Maximum (mm)			
**Orientation**	**Neuroanatomical label**	**BA**	***x***	***y***	***z***
**Delta**					
**Left**	Anterior cingulate	24	−5	35	10
**Left medial**	Medial frontal gyrus	10	−10	45	15
**Left**	Precentral gyrus	4	−35	−25	55
**Left**	Postcentral gyrus	3	−35	−25	50
**Right**	Superior parietal lobule	7	40	−60	50
**Right**	Precuneus	7	25	−75	50
**Right**	Angular gyrus	39	50	−70	30
**Right**	Inferior parietal lobule	40	40	−60	45
**Right middle**	Middle temporal gyrus	39	50	−75	25

For each significant region, the coordinates of the voxel corresponding to the maximum value (MNI, Montreal Neurological Institute) were obtained.

## Data Availability

The data that support the findings of this study are available on request from the corresponding authors. The data are not publicly available due to privacy and ethical restrictions.
